# Novel Therapeutic Strategies for Atopic Dermatitis: Biomarker Modulation and Clinical Implications. A Systematic Review

**DOI:** 10.1007/s12016-025-09129-z

**Published:** 2026-01-29

**Authors:** Noelia Moreiras-Arias, Juan José Nieto-Fontarigo, Francisco Javier Salgado, Daniel González-Vilas, Carmen Paredes-Suárez, Enma Combo-García, Carmen Rodríguez-Otero, Ángeles Flórez

**Affiliations:** 1 Department of Dermatology, Complexo Hospitalario Universitario de Santiago de Compostela, Santiago de Compostela, 15706 Spain; 2 University of Santiago de Compostela, Santiago de Compostela, 15705 Spain; 3 BioLympho Research Group, Department of Biochemistry and Molecular Biology, Faculty of Biology-Biological Research Centre (CIBUS), University of Santiago de Compostela, Santiago de Compostela, 15782 Spain; 4 Translational Research in Airway Diseases Group (TRIAD), Health Research Institute of Santiago de Compostela (IDIS), Santiago de Compostela, 15706 Spain; 5 Bibliosaúde. Servicio Gallego de Salud (Sergas), Santiago de Compostela, Spain

**Keywords:** Atopic dermatitis, Biomarkers, Biologic therapy, JAK inhibitors, Omics

## Abstract

Advances in the understanding of atopic dermatitis (AD) pathogenesis have driven the development of innovative systemic therapies targeting key immunologic pathways. This systematic review summarizes current evidence on the impact of biologic agents, Janus kinase (JAK) inhibitors, and other emerging treatments on AD-related biomarkers and their correlation with clinical outcomes. A comprehensive literature search was conducted across PubMed, Embase, Scopus, and Web of Science for studies published between 2014 and 2024. Eighty studies met the inclusion criteria. Dupilumab was the most extensively investigated therapy, followed by tralokinumab, JAK inhibitors, and novel agents such as amlitelimab, stapokibart, and tezepelumab. Across drug classes, consistent reductions in CCL17/TARC, LDH, and total IgE levels were observed, generally paralleling clinical improvement in EASI and SCORAD scores. Transcriptomic and proteomic analyses revealed normalization of Th2/Th22 inflammatory signatures and restoration of barrier-related gene expression, while microbiome studies showed a reduction in *Staphylococcus aureus* colonization. Despite these advances, the heterogeneity of study designs and analytical techniques limits the comparability of results. CCL17 and LDH currently represent the most reliable biomarkers associated with disease severity and treatment response, although their limited specificity restricts clinical applicability. Future research should aim to validate integrated biomarker panels combining immunologic, transcriptomic, and microbiomic data to enable precision medicine approaches in atopic dermatitis management.

**Supplementary Information:**

The online version contains supplementary material available at 10.1007/s12016-025-09129-z.

## Introduction

Atopic dermatitis (AD) is the most common chronic inflammatory skin disease worldwide, with an increasing prevalence that varies depending on the region and population under study [1–4]. It is estimated that AD affects approximately 20% of children and 10% of adults [1]. It is a heterogeneous disorder that is diagnosed clinically based on the presence of xerosis, intense pruritus and recurrent eczematous lesions [1–4]. Both clinical manifestations and location of the lesions vary according to age [4]. AD is frequently associated to multiple atopic comorbidities, including food allergy, asthma, allergic rhinoconjunctivitis, nasal polyposis, and eosinophilic esophagitis, which together contribute to a marked impairment in the quality of life (QoL) of patients and their families [1, 4–6]. The extent and severity of skin involvement, as well as the intensity of symptoms and patient reported outcomes (PRO) are assessed using standardized scoring systems such as the Eczema Area and Severity Index (EASI), SCORing Atopic Dermatitis (SCORAD), Investigator Global Assessment (IGA), Numerical Rating Scale for Itching (NRS-itching), Numerical Rating Scale for Sleep (NRS-sleep) and Dermatology Life Quality Index (DLQI) [2].

AD pathophysiology is complex and multifactorial, involving genetic susceptibility, epidermal barrier dysfunction, microbiome alterations, and immune dysregulation [1–10]. Mutations in the filaggrin gene (FLG, a fundamental structural protein of the stratum corneum), and less frequently in other related proteins (claudins, serine protease inhibitor LETK1, kallikreins) lead to impaired barrier function [1, 2, 10]. As a result, transepidermal water loss (TEWL) increases, skin pH is altered, and*Staphylococcus aureus(S. aureus*) colonization is promoted, further disturbing the cutaneous microbiome [1, 2, 10]. A defective barrier facilitates the penetration of allergens and irritants, triggering keratinocytes and epidermal dendritic cells to release proinflammatory cytokines (thymic stromal lymphopoietin/TSLP, IL-1, IL-25, and IL-33). These alarmins recruit and activate both innate and adaptive immune cells, including macrophages, mast cells, eosinophils, basophils, NK cells, and B and T lymphocytes [2, 10]. Within this inflammatory milieu, naïve CD4⁺ T cells differentiate predominantly into T helper (Th) type 2 (Th2) cells, producing IL-4, IL-5, IL-13, and IL-31, cytokines considered central to AD pathogenesis. To a lesser extent, differentiation toward Th1 (IFN-γ), Th17 (IL-17), and Th22 (IL-22) subsets also occurs [2, 10].

Advances in the understanding of AD pathogenesis have driven the development of innovative therapies that are reshaping clinical management. These new strategies target the hyperactivated Th2 immune response, either through the extracellular blockade of key cytokines (IL-4, IL-13, IL-31) or the intracellular inhibition of JAK-STAT signaling pathway [10–12]. Biologic agents provide selective extracellular modulation. Dupilumab, the first biologic agent approved for the treatment of moderate-to-severe AD, is a monoclonal antibody (mAb) directed against the α-subunit of the IL-4 receptor (IL-4Rα), thereby simultaneously inhibiting IL-4 and IL-13 signaling [11]. Subsequently, tralokinumab, lebrikizumab (both targeting IL-13) and nemolizumab (directed to IL-31 receptor), have been approved [11]. In contrast, Janus kinase (JAK) inhibitors act intracellularly, broadly suppressing the downstream signaling of multiple cytokine receptors that utilize JAK/STAT pathway. Currently approved JAK inhibitors for moderate-to-severe AD are upadacitinib and abrocitinib (selective JAK1 inhibitors), and baricitinib (JAK1/JAK2 inhibitor) [11]. Beyond these, emerging molecules with novel mechanisms of action are currently under investigation, including OX40/OX40-ligand inhibitors (rocatinlimab, amlitelimab, telazorlimab), dual JAK/spleen tyrosine kinase (JAK/SYK) inhibitors, new antibodies against IL-4Rα, and TSLP mAbs such as tezepelumab [11, 12].

Given this therapeutic expansion and the complex pathophysiology of AD, identifying predictive and response biomarkers is crucial to guide treatment selection, monitor efficacy, and optimize outcomes, ultimately advancing toward personalized medicine. Among the most extensively studied biomarkers are immunoglobulin E (IgE), lactate dehydrogenase (LDH), various chemokines (CCL17/ initially named as thymus- and activation-regulated chemokine/TARC, CCL18/pulmonary and activation-regulated chemokine/PARC, CCL22/macrophage derived chemokine/MDC, and CCL26/eotaxin-3), and interleukins (sIL-2r, IL-13, IL-22, IL-31), all showing promising preliminary results [7, 9]. While most of these markers have been primarily used to monitor disease activity, their potential as predictive or response biomarkers for therapy remains largely unestablished. A systematic review of these biomarkers is therefore essential to understand how novel systemic therapies influence the AD biomarker landscape.

## Methods

The main objective of this systematic review was to evaluate the effect of biologic therapies, JAK inhibitors, and other emerging drugs for AD on existing disease-related biomarkers.

 Keywords related to AD and novel therapies were selected, including*atopic dermatitis, biologic therapy, JAK inhibitor, dupilumab, tralokinumab, lebrikizumab, upadacitinib, baricitinib, abrocitinib, OX40, rocatinlimab, telazorlimab, amlitelimab, nemolizumab,* and *stapokibart*. A literature search was conducted in four databases (Embase (via Ovid), PubMed, Scopus, and Web of Science) using strategies tailored to each database through controlled vocabulary and free-text terms (available as supplementary material).

 Eligible studies included clinical trials, cohort studies, and case series published between 2014 and 2024 involving adult and/or pediatric patients with AD treated with systemic innovative therapies that assessed their effects on disease-related biomarkers. Animal studies, case reports, commentaries, conference abstracts, and studies limited to topical treatments were excluded. No language restrictions were applied.

 The retrieved records were imported into the Rayyan reference manager for duplicate detection and title/abstract screening. Two reviewers independently performed the screening process, and any discrepancies were resolved by a third reviewer.

Risk of bias was assessed using the Joanna Briggs Institute (JBI) critical appraisal checklists, applying the tool most appropriate for each study design. The overall risk was classified as low, moderate, or high according to the number and relevance of criteria met [13].

This systematic review was reported in accordance with the PRISMA (Preferred Reporting Items for Systematic Reviews and Meta-Analyses) guidelines and registered in PROSPERO (ID: CRD420251061934) [14].

 Due to the heterogeneity of the included studies (population, interventions, measurement methods, and biomarker units), results were synthesized narratively. We systematically described changes in key biomarkers, reported statistical significance, and their correlation with clinical outcomes. Extracted data are summarized in tables outlining study characteristics, evaluated biomarkers, and main findings.

## Results

### Study Selection and Characteristics

**Fig. 1:**
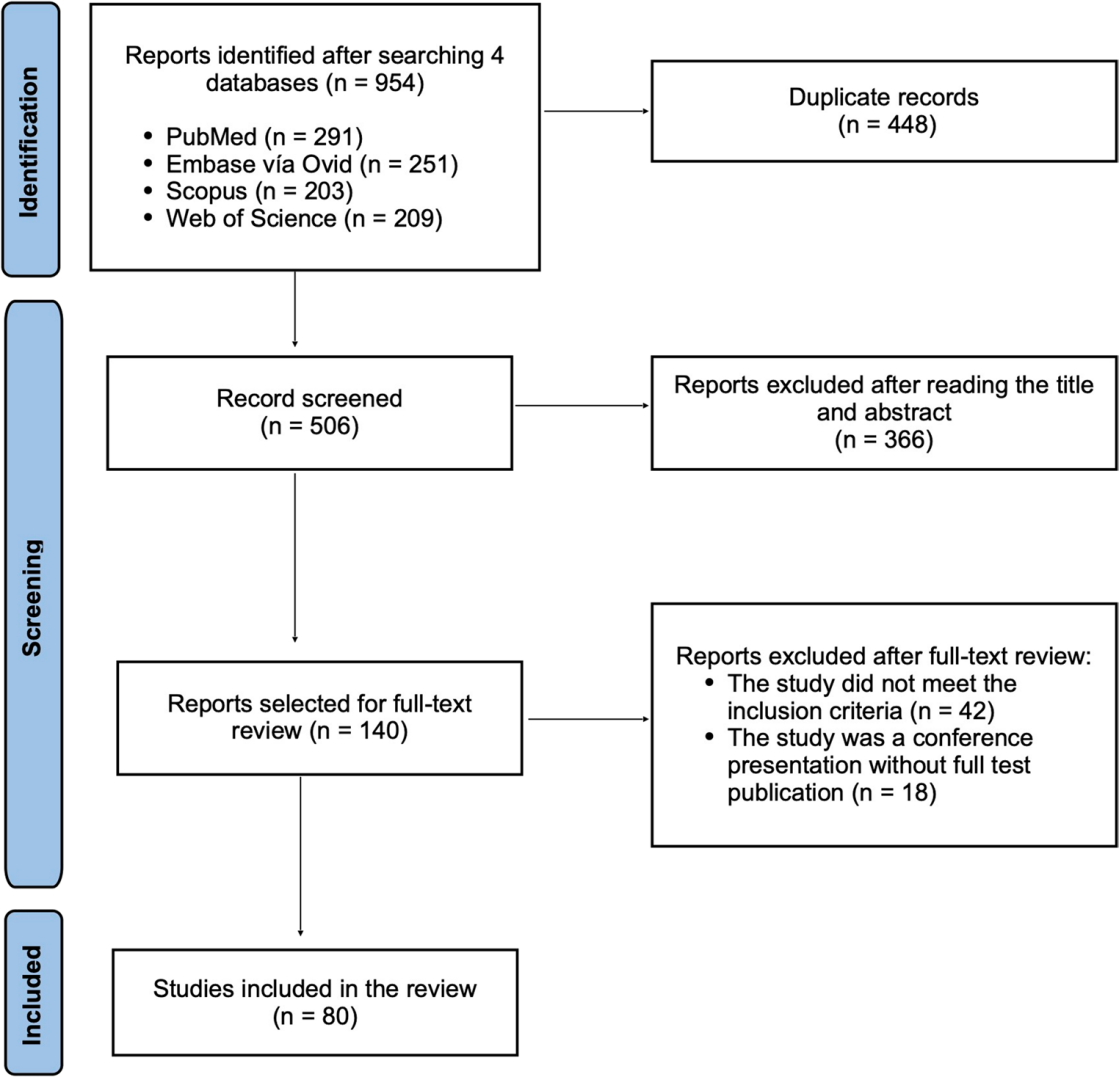
PRISMA flow diagram detailing the procedure used to select the publications included in the systematic review

A total of 955 references were identified. After removing duplicates, 507 records were screened by title and abstract. Full-text review was performed for 140 articles, and 80 studies met the inclusion criteria (Figure 1).

**Fig. 2:**
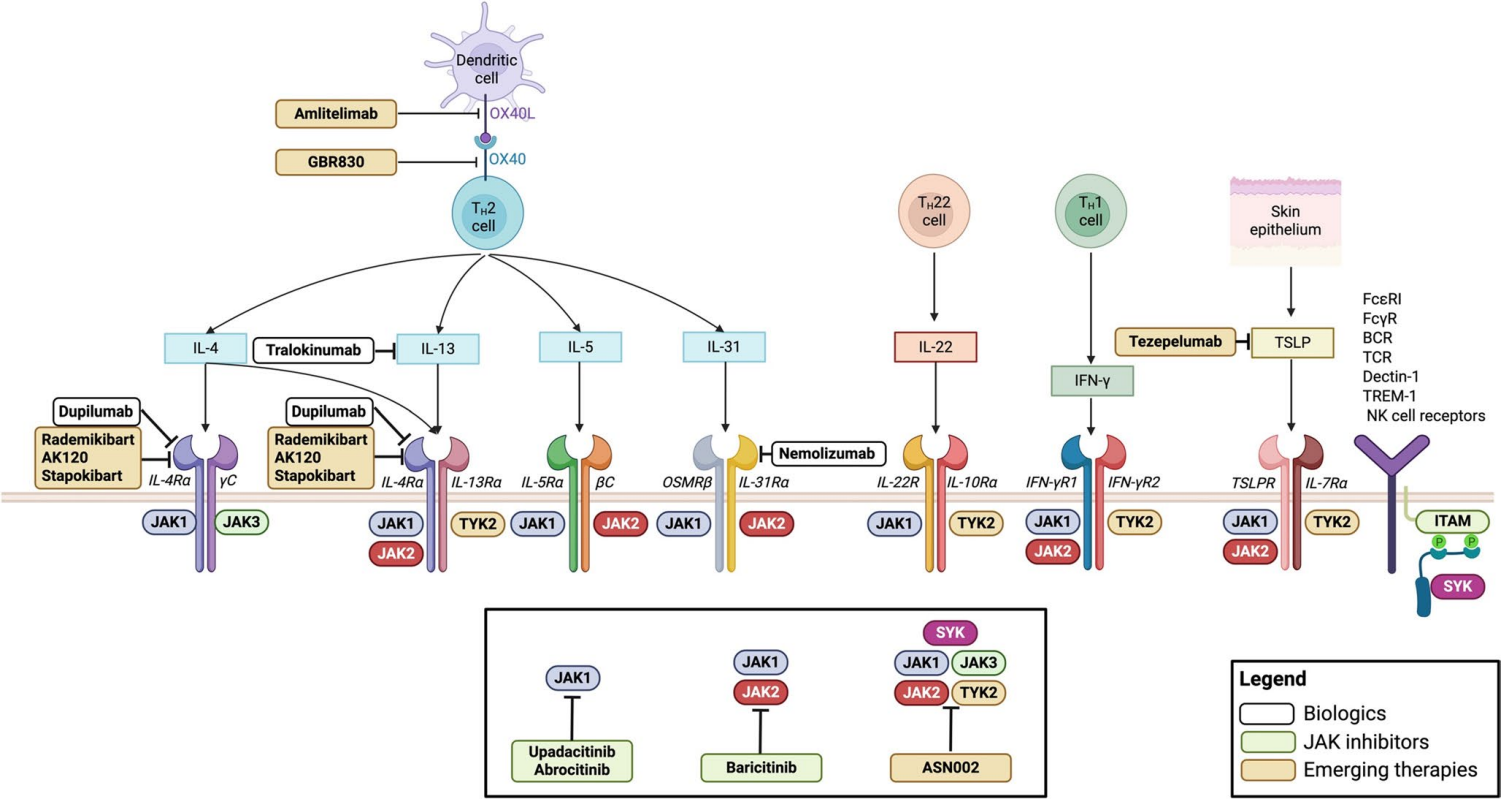
Overview of biomarker modulation and mechanistic effects of systemic therapies in AD.Targets for specific therapies included in the present review are depicted. Biologics under use in clinical settings are shown in white, whereas JAK inhibitors in green. Emerging therapies under development are shown in yellow boxes

 Biologic agents were investigated in 63 publications (Table 1), most frequently dupilumab (N=58), followed by tralokinumab (N=4) and nemolizumab (N=1) [15–77]. JAK inhibitors were evaluated in 9 studies comprising upadacitinib (N=5) abrocitinib (N=2) and baricitinib (N=2) (Table 2) [78–86]. Emerging molecules were analyzed in 8 studies (Table 3), including OX40/OX40-ligand inhibitors (GBR830 and amlitelimab, N=2), an oral JAK/SYK inhibitor (ASN002, N=2), a TSLP inhibitor (tezepelumab, N=1), and several IL-4Rα inhibitors (rademikibart, AKN120, and stapokibart, N=3) [87–94]. No eligible studies were found for lebrikizumab or rocatinlimab. Results on emerging therapies need to be interpreted cautiously due to the early-stage nature of these data (phase I/II trials). An overview of all systemic therapies evaluated is presented in Figure 2.

**Table 1:** Studies on biological therapies

Treatment	Year and authors	Study design, population, and duration	Biomarkers	Results	Correlation between biomarkers and clinical scales	Risk of bias
Dupilumab	2014 Beck et al. [15]	4 randomized double-blind placebo-controlled CT 4 weeks; *n* = 30 4 weeks; *n* =37 12 weeks; *n* = 109 12 weeks; *n* = 31 >18 years old	Serum: eosinophil count, CCL17 and IgE. Skin biopsy (lesional skin): gene expression profiles and K16.	Reduction in CCL17 and IgE levels. Improvement in the transcriptomic profile of lesional skin. Dose-dependent reduction in K16 expression.	CCL17 reduction correlates with improvement in pruritus scales. No correlation with EASI.	Low
Dupilumab	2018 Guttman-Yassky et al. [16]	Randomized double-blind placebo-controlled CT 16 weeks; *n* = 54 >18 years old	Serum: CCL17, CCL18, periostin, ECP, total IgE and allergen-specific IgE. Skin biopsy (lesional and healthy skin): gene expression profiles, epidermal hyperplasia, K16, Ki67, inflammatory cells.	Suppression of CCL17, CC18, periostin, total and specific IgE. Reduced expression of genes related to Th2 inflammation, epidermal hyperplasia, T cells, dendritic cells, and Th17/22 activity. Increased expression of genes related to epidermal differentiation, barrier function, and lipid metabolism.	Improvement in clinical (EASI, SCORAD) and histological scales significantly correlate with gene expression modulation.	Moderate
Dupilumab	2019 Huang et al. [17]	Observational 1 treatment group 12 weeks; *n* = 7 >20 years old	Serum: IgE.	Reduction in IgE in most patients (6/7).	Improvement in clinical scales (EASI, NRS) in patients with reduced IgE.	High
Dupilumab	2019 Olesen et al. [18]	Prospective observational 1 treatment group 12 weeks; *n* = 43 Age 18-78 years old	Serum: LDH, IgE, and eosinophil count.	Reduction in LDH and IgE. No significant changes in eosinophil count.	Positive correlation between: Baseline EASI-baseline LDH and 3-month reduction in EASI and LDH.	Moderate
Dupilumab	2020 Ariëns et al. [19]	Prospective observational 1 treatment group 16 weeks; *n* = 35 > 18 years old	Serum: CCL18, CCL17, periostin, sIL-2Rα, TSLP, IL-4, IL-5, IL-6, IL-8, IL-12, IL-13, IL-17, IL-20, IL-21, IL-22, IL-23, TNFα, IL-10, eotaxin 1, eotaxin 3, and elastase.	Reduction in CCL17, CCL18, periostin, IL-22, eotaxin 1 and eotaxin 3. No significant changes in the remaining ones.	Not studied.	Moderate
Dupilumab	2020 Bakker et al. [20]	Prospective observational 1 treatment group 16 weeks; *n* = 25 >18 years old	Serum: CCL17, IL-22, and sIL2r.	Reduction in CCL17 and IL-22. No changes in sIL2r	A mathematical formula based on these three biomarkers can help predict severity in patients treated with dupilumab.	Moderate
Dupilumab	2020 Callewaert et al. [21]	Randomized double-blind placebo-controlled CT 16 weeks; *n* = 54 >18 years old	Skin swabs for microbiological testing. Serum: CCL17, CCL18.	Reduction in *S. Aureus* colonization and increase in skin microbiome diversity after treatment with dupilumab. Reduction in CCL17 and CCL18.	Microbiome changes correlate with reduction in CCL17, CCL18, and improvement in clinical scales (EASI).	Low
Dupilumab	2020 Ferrucci et al. [22]	Retrospective observational 1 treatment group 16 weeks; *n* = 117 >18 years old	Serum: IgE and eosinophil count.	Slight reduction in IgE after 4 weeks of treatment.	Absence of hypereosinophilia is associated with a good response.	Moderate
Dupilumab	2020 Hamilton et al. [23]	Analysis post-hoc of 6 randomized double-blind placebo-controlled CT in various pathologies (3 AD) 16 weeks; *n* = 671 16 weeks; *n* = 708 52 weeks; *n* = 740 > 18 years old	Serum: CCL17, IgE, and eosinophil count.	Reduction in CCL17 and IgE. No significant changes in eosinophil count.	Not studied.	Low
Dupilumab	2020 He et al. [24]	Prospective observational 1 treatment group 16 weeks; *n* = 26 18–65 years old	Proteomic analysis in skin tape strips (lesional and healthy skin).	Reduction of immune markers related to general inflammation (MMP12), Th2 (CCL13/CCL17), Th17/Th22 (IL-12B, CXCL1, S100A12), IL-6, IL-8, IL-17C and proteins related to atherosclerosis/cardiovascular risk (SELE/selectin, IGFBP7, CHIT1/chitotriosidase-1, AXL). Th1 chemokines CXCL9/CXCL10 remained elevated.	Not studied.	Moderate
Dupilumab	2020 Jang et al. [25]	Retrospective observational 1 treatment group 16 weeks; *n* = 101 18-50 years old	Serum: IgE, LDH, and eosinophil count.	Reduction in LDH. No significant changes in IgE or eosinophil count.	Elevated LDH levels at 16 weeks and baseline eosinophil counts are associated with poorer response to treatment.	Moderate
Dupilumab	2020 Katoh et al. [26]	Analysis post-hoc of 2 randomized double-blind placebo-controlled CT 16 weeks; *n* = 102 52 weeks; *n* = 117 > 18 years old	Serum: CCL17 and IgE.	Rapid reduction of CCL17 and slow progressive reduction of IgE.	Not studied.	Moderate
Dupilumab	2020 Yamauchi et al. [27]	Retrospective observational 1 treatment group 32 weeks; *n* = 40 19-77 years old	Serum: CCL17, IgE, LDH and eosinophil count.	Reduction in CCL17, IgE, LDH, and eosinophil count.	Not studied.	Moderate
Dupilumab	2021 Bakker et al. [28]	Prospective observational 1 treatment group 52 weeks; *n* = 10 >18 years old	Serum and skin biopsy: study of T lymphocyte populations.	Reduction in the proportion of CD4 T cells that migrate to the skin and produce Th2/Th22 cytokines. No significant changes in T lymphocyte subpopulations after 52 weeks of treatment.	Not studied.	Moderate
Dupilumab	2021 Lee et al. [29]	Retrospective observational 1 treatment group 16 weeks; *n* = 57 >18 years old	Serum: LDH and eosinophil count.	Reduction in LDH and eosinophil count.	Correlation between reduction in EASI and reduced baseline levels of LDH and eosinophils.	Moderate
Dupilumab	2021 Mikhaylov et al. [30]	Prospective observational treatment group *n* = 18 control *n* = 17 Duration not specified > 18 years old	Skin tape strips (lesional and healthy skin): transcriptomic analysis.	Significant modulation in immune (CCL13, CCL17, CCL18) and barrier biomarkers (periplakin, FA2) with dupilumab.	Correlation between multiple biomarkers (CCL20, IL-34, FABP7) and clinical scales (EASI).	Moderate
Dupilumab	2021 Mizuno et al. [31]	Prospective observational 1 treatment group 16 weeks; *n* = 60 >18 years old	Serum: CCL17, IgE, LDH and eosinophil count.	Reduction in CCL17, LDH and IgE.	Correlation between CCL17/LDH reduction and changes in EASI.	Moderate
Dupilumab	2021 Möbus et al. [32]	Prospective observational treatment group n=39 control *n* = 31 12 weeks > 18 years old	Skin biopsy: transcriptomic analysis.	Reduction in the expression of type 2 chemokines, increased expression of IL-4, IL-5, and IL-13, as well as genes related to the skin barrier.	Not studied.	Moderate
Dupilumab	2021 Möbus et al. [33]	Prospective observational treatment group *n* = 21 control *n* = 31 12 weeks >18 years old	Skin biopsy (lesional and healthy skin): immunofluorescence and NK cell analysis (cytometry, transcriptomics).	Increase in NK cells in lesional skin and in NK cell-related gene expression. Imbalance between resting and activated cells. After dupilumab, the ratio of resting/activated cells normalizes, but alterations in the transcriptomic profile persist.	Not studied.	Moderate
Dupilumab	2022 Bae et al. [34]	Prospective observational treatment group *n* = 31 control *n* = 55 16 weeks >18 years old	Blood: Cp.	Lower Cp levels in atopic patients than in healthy controls. No changes after treatment.	Low Cp levels correlate with greater severity (EASI, DLQI).	Moderate
Dupilumab	2022 Kamphuis et al. [35]	Prospective observational < 18y 1 treatment group 28 weeks; *n* = 17 6–18 years old	Serum: CCL18, CCL17, periostin, sIL-2Rα, IL-4, IL-5, IL-13, IL-6, IL-17, IL-22, IL-23, IL-22, IL-12, IP-10, IL-1b, IL-10, GCSF, MCP1, eotaxin 1, and eotaxina 3.	Reduction in CCL17, CCL18, sIL-2Rα, periostin, and eotaxin 3.	Not studied.	Moderate
Dupilumab	2022 Kita et al. [36]	Prospective observational 1 treatment group 16 weeks; *n* = 20 17–67 years old	Serum: CCL17, IgE, and SCCA2.	Reduction in CCL17 and SCCA2.	Correlation between SCCA2 and EASI with IgE.	Moderate
Dupilumab	2022 Lee et al. [37]	Prospective observational treatment group *n* = 75 control *n* = 28 52 weeks >18 years old	Serum: adiponectin.	Low levels in patients with moderate-to-severe AD. No changes after treatment with dupilumab.	Negative correlation between pretreatment adiponectin levels and EASI and pruritus.	Moderate
Dupilumab	2022 Nettis et al. [38]	Retrospective observational 1 treatment group 16 weeks; *n* = 543 >18 years old	Serum: IgE and eosinophil count.	Reduction in IgE. No changes in eosinophil count.	Not studied.	Moderate
Dupilumab	2022 Paller et al. [39]	Randomized double-blind placebo-controlled CT 16 weeks; *n* = 161 < 18years old: 6m to 6y	Serum: LDH and eosinophil count.	Reduction in LDH and increase in eosinophil count.	Not studied.	Low
Dupilumab	2022 Spekhorst et al. [40]	Prospective observational 1 treatment group 91 weeks; *n* = 90 (from week 52, doses are spaced out) >18 years old	Serum: IL-22, CCL18, CCL17, periostin, sIL-2Rα, IL-4, IL-5, IL-13, IL-6, IL-17, IL-22, IL-23, IL-22, IL-12, IP-10, IL-1b, IL-10, GCSF, MCP1, eotaxin 1 and eotaxin 3.	Reduction in CCL17 and CCL18 at 52 weeks. Levels are subsequently maintained in all treatment subgroups.	Not studied.	Moderate
Dupilumab	2022 Tosuji et al. [41]	Retrospective observational 1 treatment group 52 weeks; *n* = 46 > 18 years old	Serum: eosinophil count.	Seven patients with elevated eosinophil count, six of whom developed conjunctivitis. Elevated eosinophil count may be a biomarker for the risk of developing conjunctivitis.	Not studied.	Moderate
Dupilumab	2022 Varandas et al. [42]	Observational 1 treatment group 52 weeks; *n* = 12 >18 years old	Serum: IL-22, IL22BP.	Elevated levels of IL-22 and reduced levels of IL-22BP in severe patients prior to treatment.	Post-treatment correlation of IL-22 with EASI and SCORAD.	High
Dupilumab	2022 Zhang et al. [43]	Prospective observational 1 treatment group 16 weeks; *n* = 33 >18 years old	Serum: IgE, eosinophil count, IL-2, IL-4, IL-6, IL-10, IL-17A, TNFα, IFNγ. Metabolomic and lipidomic study.	Reduction in IL-6, IL-10, and IFNγ. No changes in IL-2, IL-4, IL-17A, and TNFα.	Good responders associated with more changes in the metabolomic profile.	Moderate
Dupilumab	2022 Zhao et al. [44]	Randomized double-blind placebo-controlled CT 16 weeks; *n* = 82 >18 years old	Serum: CCL17, IgE, and LDH.	Reduction in CCL17, LDH, and IgE.	Not studied.	Low
Dupilumab	2023 Čelakovská et al. [45]	Prospective observational 3 groups: Dupilumab *n* = 13 No treatment *n* = 32 Healthy *n* = 30 > 18 years old	Blood: Study of B lymphocyte immunophenotype and subpopulations, CD23 and CD200 markers, T lymphocyte count (CD4, CD8), NK and regulatory T lymphocytes.	Increased neutrophils, monocytes, and eosinophils in AD. Increased CD23 and CD200 expression in AD. No differences in B and NK lymphocytes. Increased CD4 T lymphocytes and reduction in CD8 lymphocytes in patients treated with dupilumab.	Not studied.	High
Dupilumab	2023 Čelakovská et al. [46]	Prospective observational 3 groups: Dupilumab *n* = 13 No treatment *n* = 32 Healthy *n* = 30 >18 years old	Blood: total eosinophil and basophil count, CD16+ eosinophils, CD203+ basophils, and CD23 expression in B cells.	In patients treated with dupilumab: increase in eosinophil count and CD23 expression in B lymphocytes. Reduction in CD203+ basophils.	Not studied.	High
Dupilumab	2023 Kishi et al. [47]	Prospective observational 1 treatment group 16 weeks; *n* = 12 >18 years old	Serum: IgE, eosinophil count, CCL17, IL-4, IL-13, IL-22, IL-31. Skin biopsy: epidermal hyperplasia, IENF.	Reduction in CCL17, IgE, IL-22, eosinophil count, epidermal hyperplasia. Increase in IL-4. No changes in IENF, IL-13, IL-31.	Positive correlation between pruritus and CCL17, IL-22, and IgE. Positive correlation between EASI, CCL17 and IL-22.	Moderate
Dupilumab	2023 Lee et al. [48]	Prospective observational treatment group *n* = 25 control *n* = 25 18 weeks > 18 years old	Serum: ESR, CPR. Image study: ^18^F-FDG PET-CT.	Reduction in ESR and CRP levels but no significant changes in ^8^FDG uptake in major organs and aorta after treatment with dupilumab.	Not studied.	Moderate
Dupilumab	2023 Miyamoto et al. [49]	Retrospective observational 1 treatment group 16 weeks; *n* = 19 > 18 years old	Serum: Metabolomic analysis of 148 markers.	Lactic acid, alanine, glyceric acid, fumaric acid, nonanoic acid, ribose, sorbitol, and ornithine showed significant differences in responders/non-responders.	No correlation with EASI or DLQI.	Moderate
Dupilumab	2023 Montero et al. [50]	Prospective observational 1 treatment group n=32 subdivided into responders (*n* = 22) and non-responders (*n* = 10) 16 weeks > 18 years old	TEWL and SCH.	SCH increased in lesional and healthy skin. Reduction in TEWL in lesional skin.	Patients responding to dupilumab show a reduction in TEWL (lesional skin) and an increase in SCH (lesional and healthy skin).	Moderate
Dupilumab	2023 Nakahara et al. [51]	Prospective observational 1 treatment group 16 weeks; *n* = 110 >18 years old	Serum: eosinophil count, LDH, IgE, CCL17, sIL2-Rα, CCL18, CCL22, CCL26, CCL27, IL-13, IL-22, IL-24, IL-25, IL-31, IL-33, TSLP, periostin, SCCA2, and endotelin 1.	No details are provided on changes in individual biomarkers.	No correlation between baseline biomarkers and changes in EASI, but correlation with POEM (LDH, sIL-2R, CCL17, CCL22, CCL27, CCL18) and pruritus (LDH, sIL-2R, CCL17).	Moderate
Dupilumab	2023 Paller et al. [52]	Analysis post-hoc of 6 randomized double-blind placebo-controlled CT Erythrodermic patients 16 weeks; *n* = 209 >18 years old	Serum: CCL17, IgE, and LDH.	Reduction in CCL17, IgE, and LDH.	Not studied.	Low
Dupilumab	2023 Rossi et al. [53]	Retrospective observational 1 treatment group 48 weeks; *n* = 175 > 18 years old	Serum: LDH, IgE, and eosinophil count.	Reduction in LDH, IgE, and eosinophil count.	No strong correlation between biomarkers and EASI.	Moderate
Dupilumab	2023 Silverberg et al. [54]	Age-stratified analysis (</> 60 years) of 4 randomized double-blind placebo-controlled CT 16 weeks; < 60years old *n* = 2261 > 60years old *n* = 183	Serum: CCL17 and IgE.	Lower baseline IgE levels in the >60 years group. Reduction in IgE and CCL17 following treatment with dupilumab in both groups.	Not studied.	Moderate
Dupilumab	2023 Simpson et al. [55]	Randomized double-blind placebo-controlled CT 16 weeks; *n* = 71 18–65 years old	Serum: biomarkers, immune cell levels. Skin swabs: bacteriological study.	Rapid reduction in *S. Aureus* colonization (from the third day of dupilumab).	CCL17 reduction is associated with decreased *S. Aureus* colonization and improvement in clinical scales (EASI, SCORAD, IGA but not for pruritus).	Low
Dupilumab	2023 Singh et al. [56]	Retrospective observational 1 treatment group (3 subgroups: complete responders, partial responders, and non-responders) 104 weeks; *n* = 61 >18 years old	Skin biopsy: IFNγ, IL-4, IL-13, IL-22, IL-17A, IL-17F.	High levels of IL-13 and very low levels of IFNγ in responders. Partial responders and non-responders had reduced levels of IL-13 and IFNγ expression.	Elevated IL-13 is associated with a good response (unspecified scales). Non-responders have lower levels of IL-13 and higher levels of type 1/3 cytokines.	Moderate
Dupilumab	2023 Wang et al. [57]	Retrospective observational 1 treatment group 16 weeks; *n* = 34 < 18 years old	Serum: IL-10, CCL18, IL-4, CD25, CCL17, IL-21, IL-18, IL-36b, IL-1b, IL-5, IL-6, IL-17A, TSLP, CCL26, periostin, IL-12p70, CCL11, TNFα, and IFNγ.	Reduction in IL-10, CCL18, IL-4, CD25, CCL17/CCL17, IL-21, IL-18, IL-36b, TNFα, IL-1b, IL-5, IL-6, and IL-17A. Increase in TSLP. No change in other biomarkers.	Reduction in CCL17, IL5, and CD25 is associated with improvement in SCORAD, EASI, and pruritus.	Moderate
Dupilumab	2023 Wu et al. [58]	Prospective observational treatment group *n* = 125 control *n* = 65 16 weeks >12 years old	Serum: IgE, eosinophil count, and panel of 24 cytokines/chemokines (IL12, IL-18, TNFα, IFNγ, CCL18, CCL17, IL-4, IL-5, IL-13, TSLP, IL-31, IL-33, IL-22, IL-17A, IL-23, IL-6, IL-21, IL-10, IL2-Rα, eotaxin 3, periostin, eotaxin 1, IL-1β, IL-36β)	Reduction in CCL17, CCL18, IL-4, IL-5, IL-31, IL-33, IL-12, IL-18, TNFα, IL-6, IL-17A, IL2-Rα, and periostin. Increase in IL-13.	Good responders to dupilumab have elevated baseline levels of IL2-Rα, IL-31, and IL-36 and a reduction after treatment.	Moderate
Dupilumab	2024 Beck et al. [59]	Retrospective analysis of 3 randomized double-blind placebo-controlled CT Pediatric population; 6m-5y *n* = 62 6–11y *n* = 367 12–17y *n* = 251 16 weeks	Serum: CCL17, IgE, LDH, and eosinophil count.	Reduction in CCL17, IgE, and LDH. No changes in eosinophil count.	Not studied.	Low
Dupilumab	2024 Čelakovská et al. [60]	Prospective observational 3 groups: Dupilumab *n* = 13 No treatment n=32 Healthy *n* = 30 > 18 years old	Blood: total eosinophil and basophil count, CD16+ eosinophils, CD203+ basophils, and CD200 expression in B cells	In patients treated with dupilumab: increase in eosinophil count and CD200 expression in B lymphocytes Reduction in CD203+ basophils	Not studied	High
Dupilumab	2024 Čelakovská et al. [61]	Prospective observational 3 groups: Dupilumab *n* = 24 No treatment *n* = 29 Healthy *n* = 40 > 14 years old	Blood: immunological profile study during the pollen season (June-August).	In patients treated with dupilumab: Increase in eosinophils, reduction in CD8+ T lymphocytes, normalization of regulatory T lymphocytes and memory B lymphocytes, but persistence of immunological alterations related to dysregulation and hyperactivation of B lymphocytes.	Not studied.	Moderate
Dupilumab	2024 Dekkers et al. [62]	Prospective observational 1 treatment group 16 weeks; *n* = 127 >18 years old	Serum: study of the proteomic profile to define two subgroups: Th2 dominant and non-dominant.	No protein predicting response to dupilumab was identified.	Patients with a dominant Th2 profile do not respond better than those without a dominant profile.	Moderate
Dupilumab	2024 Kenney et al. [63]	Data analysis from a randomized double-blind placebo-controlled CT 16 weeks; *n* = 71 18–75 years old	Skin biopsies of lesional and healthy skin: transcriptomic study (CERS1). Skin swabs: microbiological studies.	CERS1 expression is associated with increased colonization by *S. Aureus* on lesional and non-lesional skin and reduced production of long-chain fatty acids. Rapid reduction in CERS1 expression following dupilumab.	CERS1 expression is associated with greater severity (EASI/SCORAD).	Moderate
Dupilumab	2024 Kido-Nakahara et al. [64]	Prospective observational 1 treatment group 16 weeks; *n* = 110 > 18 years old	Serum: eosinophil count, LDH, sIL-2R, CCL17, CCL18, CCL22, CCL26, CCL27, IL-13, IL-22, IL-24, IL-25, IL-31, IL-33, TSLP, periostin, SCCA2, and ET-1.	Elevated baseline levels of LDH and periostin were associated with poorer response.	No correlation between baseline biomarker levels and EASI, POEM, NRS-itch.	Moderate
Dupilumab	2024 Kim et al. [65]	Retrospective observational 3 groups: SCIT *n* = 20 dupilumab *n* = 14 SCIT + dupilumab *n* = 14 76 weeks > 18 years old	Serum: specific IgE D1/D2, specific Ig G4 D1/D2.	Reduction in specific IgE with significant differences in favor of the groups receiving dupilumab monotherapy or combined with SCIT. Increase in specific IgG4 in the groups receiving SCIT monotherapy or combined with dupilumab.	No statistically significant differences in EASI between the different treatment groups.	High
Dupilumab	2024 Limão et al. [66]	Retrospective observational 1 treatment group 52 weeks; *n* = 33 > 18 years old	Serum: IgE, LDH, CPR eosinophil count and airborne allergens sensitization (cat epithelium, *Dermatophagoides pteronyssinus, Dermatophagoides farinae, Lepidoglyphus destructory, Phleum pratense, Olea europaea, Parietaria judaica*, and dog epithelium).	Reduction in IgE, LDH, and specific IgE against cat epithelium, *Dermatophagoides pteronyssinus, Dermatophagoides farinae, Lepidoglyphus destructory,* and *Phleum pratense.* No significant changes in CRP, eosinophils, and IgE against *Olea europaea, Parietaria judaica*, and dog epithelium.	Not studied.	Moderate
Dupilumab	2024 Mitroi et al. [67]	Prospective observational Treatment group *n* = 22 control *n* = 20 12 weeks >18 years old	Serum: IL-4 and IL-13.	Elevated baseline levels of IL-4 and IL-13 in AD compared to controls. Increase in IL-4 and IL-13 after dupilumab.	No statistically significant data.	Moderate
Dupilumab	2024 Monedeiro et al. [68]	Prospective observational 1 treatment group 16 weeks; *n* = 8 18–65 years old	Blood: ICP and inflammatory mediators. Interstitial dermal fluid from lesional and healthy skin: ICP, inflammatory mediators, metabolomics, and miRNA. Lesional skin biopsy: miRNA.	Reduction of immune cells and inflammatory mediators. Change in lipid composition with an increase in long-chain or very long-chain fatty acids.	Correlation between improvement in clinical scales (EASI, SCORAD, IGA) and increase in long-chain fatty acids.	Moderate
Dupilumab	2024 Pažur et al. [69]	Prospective observational 3 groups: Dupilumab *n* = 50 Healthy *n* = 39 Psoriasis *n* = 15 24 weeks > 18 years old	Serum: proteomic analysis y miRNA. Skin swabs: microbiological studies.	Reduction in CCL17, CCL13, CCL22, E-selectin, and colonization by *S. Aureus.* Increase in BNDF. Neither baseline microbial composition nor miRNA pattern was associated with differences in response to dupilumab.	Positive correlation SCORAD, BSA, pruritus, and CCL17, CCL13, E-selectin. Negative correlation BSA and BDNF.	Low
Dupilumab	2024 Raimondo et al. [70]	Prospective observational Treatment group *n* = 25 y control *n* = 15 104 weeks >18 years old	Urine: biopyrin. Serum: IgE, CPR.	Increased baseline levels in AD. Progressive decrease during treatment, maintained at 52 and 104 weeks.	Positive correlation between biopyrin, EASI, IgE, and CRP.	Moderate
Dupilumab	2024 Scala et al. [71]	Prospective observational 1 treatment group 52 weeks; *n* = 17 >18 years old	Serum: specific IgE against HEMAs.	IgE against HEMAs more common in severe AD.	Poor response is associated with IgE against enolase and inversely with IgE against manganese superoxide dismutase (MnSOD) and the NPC2 family.	Moderate
Dupilumab	2024 Stölzl et al. [72]	Prospective observational 1 treatment group 24 weeks; *n* = 14 < 18 years old	Skin tape strips (lesional and healthy skin): proteomic analysis of 21 biomarkers.	Most significant reduction in fibronectin, IL-8, and S100A9 levels.	Not studied.	Moderate
Nemolizumab	2022 Sidbury et al. [73]	Prospective observational 1 treatment group 20 weeks; *n* = 20 < 18 years old	Serum and skin tape strips (lesional and healthy skin): analysis of 30 biomarkers.	Reduction in the expression of CCL17, CCL20, CCL22, CCL27, VEGF, IL-1RA, and CCL18.	No correlation between serum biomarkers and clinical scales.	Moderate
Tralokinumab	2019 Wollenberg et al. [74]	Randomized double-blind placebo-controlled CT 12 weeks; *n* = 204 18-75 years old	Serum: DPP-4, periostin, CCL17, and IgE. Subgroups were established based on high or low results for each biomarker.	Reduction in periostin, CCL17, and IgE Increase in DPP-4.	Subgroups with high levels of DPP-4 and periostin showed better response (EASI).	Low
Tralokinumab	2024 Dekkers et al. [75]	Prospective observational 1 treatment group 28 weeks; *n* = 84 > 18 years old	Serum: 18 biomarkers and IgE.	Reduction in IgE in subgroup of patients who had not previously received dupilumab Reduction in CCL17, CCL18 IL-4, IL-5, and periostin remained stable Increase in IL-13	Not studied	Moderate
Tralokinumab	2024 Guttman-Yassky et al. [76]	Data analysis from 2 randomized double-blind placebo-controlled CT ECZTRA 1 52 weeks ECZTEND 52 weeks *n* (serum)= 299 (223 tralokinumab; 76 placebo) *n* (skin biopsy) = 35 tralokinumab; 5 placebo; 13 tralokinumab after 2y > 18 years old	Serum: CCL17, IL-22, periostin and IgE. Skin biopsy: transcriptomic profile and immunohistochemistry.	Reduction in CCL17, IL-22, periostin, IgE, and skin thickness. Improvement in the transcriptomic profile with reduction in the expression of genes involved in Th1/Th2/Th17/Th22 inflammatory pathways and systemic inflammation. Increase in the expression of genes related to barrier function.	Reduction in CCL17, IL-22, and periostin correlates with reduction in EASI at 16 weeks.	Moderate
Tralokinumab	2024 Sander et al. [77]	Prospective observational 1 treatment group 16 weeks; *n* = 16 > 18 years old	TEWL and SCH analysis. Skin swabs: microbiological studies. Skin biopsy (lesional and healthy skin): histopathological and immunohistochemical study. Skin tape strips (lesional and healthy skin): proteomic analysis.	Reduction in TEWL, certain inflammatory biomarkers and those related to skin barrier dysfunction (CCL17, fibronectin, IL-8), spongiosis, K16 expression, skin thickening, and colonization by *S. aureus.* Increase in stratum corneum hydration.	Not studied.	Moderate

^18^*F-FDG PET-CT* Positron emission tomography with 18F-labeled fluorodeoxyglucose and computed tomography; *AD* atopic dermatitis; *BDNF* brain-derived neurotrophic factor; BSA: body surface area; *CERS1* ceramide synthase 1; *CPR* C-reactive protein; *CT* clinical trial; *D1* Dermatophagoides pteronyssinus; *D2* Dermatophagoides farinae; *DLQI* quality of life index in dermatology; *DPP-4* dipeptidyl peptidase 4; *EASI* Eczema area and severity index; *ECP* eosinophilic cationic protein; *ESR* erythrocyte sedimentation rate; *GCSF* granulocyte colony-stimulating factor; *HEMAs* human-homologous exogenous molecular allergens; *ICP* immune cell populations; *Ig* immunoglobulin; *IGA* investigator's global assessment; *IL* interleukin; *IFNγ* gamma interferon; *K16* keratin 16; *LDH* lactate dehydrogenase; *m* months; *miRNA* microRNA; *MCP-1* monocyte chemotactic protein 1; *CCL18* pulmonary and activation-regulated chemokine; *SCIT* subcutaneous immunotherapy; *sIL2r* soluble IL-2 receptor; *SCORAD* SCORing Atopic Dermatitis; *SCH* stratum cornea hydration; *CCL17* thymus and activation-regulated chemokine; *TEWL* transepidermal water loss; *TNFα* alpha tumoral necrosis factor; *TSLP* thymic stromal lymphopoietin; *y* years

**Table 2:** Studies on JAK inhibitors

Treatment	Year and authors	Study design, population, and duration	Biomarkers	Results	Correlation between biomarkers and clinical scales	Risk of bias
Abrocitinib	2022 Gooderham et al. [78]	Post-hoc analysis of 1 randomized double-blind placebo-controlled CT 4 weeks after stopping treatment 16 weeks; *n* = 267 > 18 years old	Serum: CCL17, IL-31, CPR, and eosinophil count.	Increase in all biomarkers to near baseline levels 4 weeks after discontinuing abrocitinib, with CRP and eosinophil count being the most affected.	Not studied.	Low
Abrocitinib	2024 Guttman-Yassky et al. [79]	Randomized double-blind placebo-controlled CT (variable dose 100mg/200mg) 12 weeks; *n* = 46 > 18 years old	Skin biopsy (lesional and healthy skin): biomarker study (MMP-12, K16, CCL17, CCL18, CCL26, S100A8, S100A9, and S100A1), gene expression analysis, and changes in epidermal hyperplasia.	Reduction in MMP-12, K16, S100A8, S100A9, and S100A1 with both doses and in a dose-dependent pattern. Reduction in CCL17 and CCL18 with the 200 mg dose. No changes in CCL26.	Significant correlation between changes in EASI, IGA, PP-NRS, and certain biomarkers (K16, CCL18, S100A8, S100A9, S100A1).	Low
Baricitinib	2019 Konrad et al. [80]	Randomized double-blind placebo-controlled CT 16 weeks; *n* = 124 >18 years old	Serum: IL-19.	Reduction in IL-19.	Significant correlation between IL-19 and changes in EASI.	Moderate
Baricitinib	2024 Watanabe et al. [81]	Retrospective observational 1 treatment group 12 weeks; *n* = 30 15-54 years old	Serum: CCL17, LDH, IgE, and eosinophil count.	No significant changes were detected in any biomarker.	No statistically significant data.	Moderate
Upadacitinib	2022 Hagino et al. [82]	Retrospective observational 1 treatment group 12 weeks; *n* = 31 > 12years old	Serum: CCL17, LDH, IgE, and eosinophil count.	Reduction in CCL17, LDH, IgE, and eosinophil count.	Correlation between reduction in eosinophil count and reduction in EASI.	Moderate
Upadacitinib	2023 Hagino et al. [83]	Prospective observational 1 treatment group 24 weeks; *n* = 65 > 12years old	Serum: eosinophil-to-lymphocyte ratio, monocyte-to-lymphocyte ratio, neutrophil-to-lymphocyte ratio, and platelet-to-lymphocyte ratio.	Sustained reduction in the eosinophil-lymphocyte and neutrophil-lymphocyte ratios until week 24. Initial reduction in the monocyte-lymphocyte and platelet-lymphocyte ratios, but return to baseline levels from week 12 onwards.	Correlation between reduction in eosinophil-lymphocyte ratio and reduction in EASI and PP-NRS.	Moderate
Upadacitinib	2023 Li et al. [84]	Prospective observational 1 treatment group 24 weeks; *n* = 25 >12years old	Blood: 25 biomarkers, neutrophils, eosinophils, basophils, monocytes, T lymphocytes, B lymphocytes, and NK cells.	Reduction in eosinophils, neutrophils, Th1, Th2, Th17, Th22 markers, CD4+ IL-22 T cells, and IL-22.	Not studied.	Moderate
Upadacitinib	2024 Hagino et al. [85]	Retrospective observational 1 treatment group 48 weeks; *n* = 283 >12years old	Serum: CCL17, LDH, IgE, and eosinophil count.	Reduction in CCL17, LDH, IgE, and eosinophil count.	Correlation between reduction in eosinophil count and reduction in EASI and PP-NRS.	Moderate
Upadacitinib	2024 Koga et al. [86]	Observational 1 treatment group 96 weeks; *n* = 14 12–19 years old	Serum: CCL17, IgE, and eosinophil count.	Increase in CCL17 and IgE until week 24, followed by a reduction. No changes in eosinophil count.	Not studied.	Moderate

*CPR* C-reactive protein; *CT* clinical trial; *DPP-4* dipeptidyl peptidase 4; *EASI* Eczema area and severity index; *Ig* immunoglobulin; *IGA* investigator's global assessment; *IL* interleukin; *K16* keratin 16; *LDH* lactate dehydrogenase; *m* months; *miRNA* microRNA; *MMP* metalloproteinase; *NK* natural killer cells; *PP-NRS* peak pruritus numerical rating scale; *CCL17* thymus and activation-regulated chemokine; *y* years

**Table 3:** Studies on novel therapies under investigation

Treatment	Year and authors	Study design, population, and duration	Biomarkers	Results	Correlation between biomarkers and clinical scales	Risk of bias
GBR830 (OX40 inhibitor)	2019 Guttman-Yassky et al. [87]	Randomized double-blind placebo-controlled CT 85 days; *n* = 64 > 18 years old	Skin biopsy: inflammatory markers and epidermal hyperplasia study.	Significant reduction in mRNA expression of Th1 (IFNγ, CXCL10), Th2 (IL-31, CCL11, CCL17), and Th17/Th22 (IL-23p19, IL-8, S100A12) markers in lesional skin. Reduction in OX40 T cells and OX40 dendritic cells. Reduction in epidermal hyperplasia markers.	Not studied.	Low
ASN002 (JAK/SYK inhibitor)	2019 Bissonnette et al. [88]	Randomized double-blind placebo-controlled CT 4 weeks; *n* = 36 >18 years old	Serum: several panels of inflammatory markers.	Reduction of biomarkers related to innate immunity, T and B lymphocytes, Th1, Th2, Th17, Th22 pathways, and atherosclerosis (E-selectin).	Not studied.	Low
ASN002 (JAK/SYK inhibitor)	2019 Pavel et al. [89]	Randomized double-blind placebo-controlled CT 4 weeks; *n* = 36 >18 years old	Skin biopsy (lesional and healthy skin): study of inflammatory markers, transcriptomic profile, and barrier function.	Rapid changes in the transcriptomic profile by suppressing key inflammatory pathways. Improvement in barrier function markers.	Correlation of clinical scales (EASI, BSA) with various combinations of biomarkers. Day 15: EASI correlates with MMP12, IFNγ, IL-13, IL-5, CCL13, CCL17, CCL22, CCL26, IL-22 and CCL20. BSA correlates with MMP12, IL-13, CCL22, CCL17, IL-22 and CCL20. Day 29: EASI correlates with IL-6, IL-15, CXCL10, S100A7, S100A9, and S100A8. BSA correlates with IL-31, IL12B/IL-12p40, IL-9, IL-22, IL-13, MMP12, IL-19 and IL-6.	Low
Tezepelumab (TSLP inhibitor)	2019 Simpson et al. [90]	Randomized double-blind placebo-controlled CT 12 weeks; *n* = 113 >18 years old	Serum: periostin, DPP-4, CCL17, and IgE.	Classification into subgroups based on the 4 biomarkers.	Better response data in patients with reduced levels of periostin and CCL17 and increased levels of DPP-4 and IgE.	Low
Rademikibart (IL-4α receptor inhibitor)	2023 Wang et al. [91]	2 randomized double-blind placebo-controlled CT 85 days; *n* = 40 11 weeks; *n* = 32 > 18 years old	Serum: CCL17, LDH, and eosinophil count.	Reduction in CCL17 and LDH. No changes in eosinophil count.	Not studied.	Low
AK120 (IL-4α receptor inhibitor)	2023 Wynne et al. [92]	Randomized double-blind placebo-controlled CT 12 weeks; *n* = 40 18–55 years old	Serum: CCL17 and IgE.	Reduction in CCL17 and IgE.	Not studied.	Low
Amlitelimab (OX40 ligand inhibitor)	2024 Weidinger et al. [93]	Randomized double-blind placebo-controlled CT 52 weeks; *n* = 388 >18 years old	Serum: CCL17, LDH, IgE, IL-13, IL-22, IL-17A, IL-31, and eosinophil count.	Reduced levels of all biomarkers tested. Levels remained low even in patients who discontinued the medication.	Not studied.	Low
Stapokibart (IL-4α receptor inhibitor)	2024 Zhang et al. [94]	2 randomized double-blind placebo-controlled CT 12 weeks n1=33 n2=39 > 18 years old	Serum: CCL17 and IgE	Reduction in CCL17 and IgE.	Not studied.	Low

*BSA* body surface area; *CPR* C-reactive protein; *CT* clinical trial; *DPP-4* dipeptidyl peptidase 4; *EASI* Eczema area and severity index; *IFNγ* gamma interferon; *Ig* immunoglobulin; *IGA* investigator's global assessment; *IL* interleukin; *K16* keratin 16; *LDH* lactate dehydrogenase; *miRNA* microRNA; *CCL17* thymus and activation-regulated chemokine; *TSLP* thymic stromal lymphopoietin

Of the 80 included studies, 25 were clinical trials [15, 16, 21, 23, 26, 39, 44, 52, 54, 55, 59, 63, 74, 76, 78–80, 87–94] and 55 were observational studies [17–20, 22, 24, 25, 27–38, 40–43, 45–51, 53, 56–58, 60–62, 64–73, 75, 77, 81–86] of which 15 included a control group [30, 32–34, 37, 45, 46, 48, 58, 60, 61, 65, 67, 69, 70].

 Information regarding age was extracted from each study to determine the representation of pediatric, adolescent, adult, and elderly populations (Tables 1–3). The vast majority of studies were conducted in adults (73 out of 80 studies; 91.2%). For dupilumab, 53 of 58 studies (91.3%) enrolled adults (two of them also included some adolescents) [15–34, 36–56, 58, 60–71], 4 studies included both children (<12 years old) and adolescents (12–18 years old) [35, 57, 59, 72], and only one study was performed exclusively in young children (<6 years old) [39]. The work in nemolizumab [73]was carried out in pediatric population, whereas all tralokinumab studies (N=4) were conducted in adults [74–77]. For JAK inhibitors, most trials were performed in adults (8/9; 89%) [78–86]. Specifically, of the five upadacitinib studies, four included some adolescents (>12 years of age) [82–85], while the remaining study was conducted exclusively in adolescents (12–19 years old) [86]. All studies evaluating emerging therapies were limited to adult populations [87–94]. Regarding elderly patients, they were included within most adult trials but were not assessed as an independent or predefined subgroup (Tables 1–3); therefore, evidence in this population remains limited.

 The biomarkers, biological samples, and analytical techniques used across included studies were highly heterogeneous. Nevertheless, several patterns were evident. In the next sections of the results, we will summarize the main findings.

### Changes in Classical Biomarkers in AD Patients Under Systemic Innovative Treatment

Classical biomarkers commonly assessed in clinical practice for AD include total IgE, lactate dehydrogenase (LDH), eosinophil counts, and CCL17; however, routine measurement of the latter is currently implemented only in Japan [7–9].

 First, all studies evaluating changes in CCL17 and LDH (48 out of 80 studies; 60%) reported a reduction in both biomarkers, regardless of the drug class (Tables 1–3) [15, 16, 18–21, 23–27, 29–32, 35, 36, 39, 40, 44, 47, 51–54, 57–59, 64, 66, 69, 73–79, 81, 82, 84–91]. Second, total IgE levels significantly decreased after dupilumab treatment in most studies (22 out of 24; 92%), except for two (Table1) [17, 25]. Three studies also reported reduction in specific IgE after dupilumab (Table1) [65, 66, 71]. Total serum IgE was also reduced with tralokinumab (3 out of 3; 100%) [74–76], or JAK1 inhibitors (i.e., upadacitinib; 3 out of 3; 100%) [82, 85],whereas no change was reported for JAK1/2 inhibitor baricitinib (Tables1 and 2) [81].Emerging therapies, including stapokibart, AKN120, and the anti-OX40L mAb amlitelimab, also had an effect on reducing IgE levels (Table3) [86, 92–94]. Finally, eosinophil counts showed variable trends. They remained stable in most dupilumab studies (9 out of 20; 45%) [15, 18, 22, 23, 25, 31, 38, 59, 66], but increased in a few (6/20; 30%) (Table 1) [39, 41, 45, 46, 60, 61]. Furthermore, Touji et al. reported seven patients with eosinophilia after dupilumab therapy, and six of them developed conjunctivitis, suggesting that elevated eosinophils may represent a potential biomarker for dupilumab-associated conjunctivitis risk [41].In contrast, upadacitinib tended to decrease eosinophil counts in most of the studies (4/5; 80%) (Table 2) [82, 84, 85]. Data are limited for the other drugs included in this review.

 Interestingly, reductions in the classical inflammatory biomarkers were associated to improvement in several clinical outcomes in 18 out 20 studies (90%), particularly with biologic therapies. For example, reductions in CCL17, LDH, IgE correlated with lower EASI scores in patients treated with dupilumab (Table 1) [15, 17, 18, 21, 25, 29, 31, 47, 57] reinforcing their relevance as indicators of dupilumab response. CCL17 was also correlated with EASI improvement in patients treated with tralokinumab [76]. Conversely, higher baseline LDH and eosinophil levels were associated with poorer dupilumab response, suggesting their potential as negative predictive biomarkers (Table 1) [25, 29]. In the case of JAK inhibitors, the reduction of eosinophil numbers was correlated with improvement in EASI and/or pruritus NRS in patients receiving upadacitinib (Table 2) [82, 83, 85].

### Changes in Inflammatory Markers and Other Soluble Proteins in Response to Systemic Innovative Therapies in AD

 Classical biomarkers consistently reflected treatment-related immunomodulation across the studied drugs and, in many cases, paralleled clinical improvement. However, these routinely measured indicators only capture part of the complex inflammatory signature of AD. To better understand the mechanistic effects of novel systemic therapies and to identify specific predictors of treatment response, several studies have evaluated broader panels of cytokines and soluble proteins. These additional biomarkers provide complementary insights into pathway-specific modulation and may help refine future predictive or response-oriented biomarker strategies.

 Beyond CCL17, frequently evaluated cytokines included CCL18, IL‑1β, IL-4, IL-5, IL-6, IL-8, IL-10, IL-12, IL-13, IL-17A/F, IL-19, IL-23, IL-36β, IFN-γ, TSLP and TNF‑α (Tables 1–3) [15–94]. In studies with dupilumab IL-4 showed variable changes whereas IL-13 remained stable or increased and IL-22 decreased reflecting the selective inhibition of Th2-mediated inflammation (Table1) [19, 24, 32, 35, 40, 43, 47, 56–58, 68]. Tralokinumab produced a slightly different profile, with both IL-4 and IL-22 decreased while IL-13 increased (Table 1) [75–77], which suggest subtle differences in Th2 pathway modulation between IL-4R and IL-13 blocking. Importantly, the rise in circulating IL-13 after IL-13 blockade does not indicate enhanced Th2 activity; rather, it reflects target engagement, as neutralizing antibodies form IL-13-antibody complexes that prolong IL-13 half-life and increase measurable serum concentrations. Despite these differences, IL‑22 reduction correlated with EASI improvement in both patients treated with dupilumab and tralokinumab [42, 47, 76] (Table 1), suggesting its potential as a T2-biologic treatment response biomarker. JAK inhibitors studies did not report consistent cytokine changes (Table 2), as expected due to their broad immunomodulatory spectrum blocking JAK/STAT signaling, and subsequently affecting multiple Th pathways (i.e., Th1, Th2, and Th22) [78–86]. However, it is important to point out the significant correlation between IL‑19 and EASI changes reported by Konrad et al. [80]. Finally, OX40/OX40L inhibitor amlitelimab selectively reduced IL-13 (Table 3) [93].

 In addition to cytokines, numerous soluble proteins with diverse biological functions and inflammatory markers were analyzed in AD patients receiving systemic therapies (Tables 1–3). Soluble proteins included adiponectin, ceruloplasmin, dipeptidyl peptidase 4 (DPP-4), elastase, eotaxin-1, eotaxin-3, periostin, and sIL-2Rα, among others [15–94]. As expected, T2 cytokine-induced proteins such as eotaxin-3 (eosinophilic chemoattractant) and periostin were generally decreased with dupilumab (Table 1) [16, 19, 58]. In tralokinumab-treated patients periostin either decreased (2 out of 3 studies; 67%) or remained stable (33%) and its reduction correlated with reduction in EASI at 16 weeks [74–76]. Other serum proteins were inversely associated with disease severity, but they did not change with dupilumab treatment. For example, lower levels of ceruloplasmin were found in patients with higher EASI or DLQI scores [34], and reduced adiponectin levels were associated with more severe disease (EASI, pruritus) [37]. However, none of these proteins changed following dupilumab therapy [34, 37].

 On the other hand, systemic inflammatory markers, including erythrocyte sedimentation rate (ESR) and C reactive protein (CRP) levels were both decreased after dupilumab therapy (Table 1), while no changes were observed in 18F-Fludeoxyglucose (^18^FDG) uptake in major organs by positron emission tomography scan and computed tomography (PET-CT) [48]. No studies specifically addressed changes in soluble proteins or inflammatory biomarkers in response to JAK inhibitors or emerging therapies.

### Molecular Profiling of Systemic Innovative Therapy in AD Using -Omic Strategies

 In recent years different non-targeted -omic studies (i.e., Transcriptomic, Proteomic, Metabolomic and Lipidomic) have been increasingly applied to detect biomarkers associated with systemic therapy. Transcriptomic analyses of lesional skin biopsies showed downregulation of several genes related to Th2 inflammation and epidermal hyperplasia, and upregulation of genes associated with barrier function and lipid metabolism in response to dupilumab treatment (Table 1) [15, 16, 68]. Targeting IL‑13 with tralokinumab demonstrated similar changes in skin transcriptomic profile, with decreased expression of genes linked to Th1/Th2/Th17/Th22 and systemic inflammation, and increased expression of barrier-related genes (Table 1) [76]. Interestingly, changes in transcriptomic profiles in response to biologics were correlated with improvement in clinical scores (e.g., EASI, SCORAD) in two studies (Table 1) [16, 76]. Abrocitinib also showed favorable results at 12 weeks, significantly and dose-dependently reducing expression of genes involved in inflammation, epidermal hyperplasia, and Th2/Th22 responses (Table 2) [79]. Moreover, several of these skin biopsy biomarkers (K16, CCL18, S100A8, S100A9, S100A1) displayed significant associations with EASI/IGA/NRS-pruritus improvement after abrocitinib therapy [79]. Short-term transcriptomic studies with GBR830 and ASN002 also demonstrated similar patterns (Table 3) [87, 89].

 Proteomic analyses using tape-stripping samples were performed in three studies with dupilumab and one with tralokinumab, revealing suppression of several immune mediators (e.g., CCL13, CCL17, or S100 proteins) with both therapies (Table 1) [24, 30, 72, 77]. Metabolomic and lipidomic studies have been only performed in dupilumab-treated patients revealing significant changes among treatment responders, including increased levels of long- and very-long-chain fatty acids [49, 63, 68]. Despite these promising findings, current evidence is limited by the small number of studies and the heterogeneity of -omic approaches. Further well-designed studies are warranted to validate these biomarkers, expand -omic analyses to other systemic therapies, and clarify their predictive value for clinical response.

### Immunophenotypic Changes in AD Patients During Systemic Innovative Therapy

Several studies by Čelakovská et al. examined the effects of dupilumab on circulating immune cell populations, including the changes in different lymphocyte subsets (B and T cells), biomarkers, and other hematologic cell subpopulations such as basophils and NK cells [45, 46, 60, 61]. Across these studies, dupilumab was associated with a decrease in circulating CD8⁺ T cells, the normalization of regulatory T cells and memory B cells, an increased expression of CD200 and CD23 in B cells (i.e., suggesting a less pro-inflammatory B cell phenotype), and a reduction in CD203⁺ basophils [45, 46, 60, 61].

 Möbus et al*.*works demonstrated increased numbers of NK cells and altered gene expression profiles in lesional skin of AD patients, along with an imbalance between resting and activated cells [33]. Strikingly, treatment with dupilumab normalized this resting/activated NK cell ratio, although transcriptomic alterations persisted [33]. Collectively, these findings suggest that the therapeutic effects of dupilumab may extend beyond cytokine modulation, promoting immunologic rebalancing via modulation of cellular activation and tolerance mechanisms. However, more supporting evidence is needed to confirm these results and to define their clinical relevance. Current studies are limited by small sample sizes, heterogeneous patient populations, and variability in sampling timepoints during therapy.

### Changes in Skin Microbiome and Barrier Function in AD Associated to Systemic Innovative Therapies

 Targeted systemic therapies, particularly biologic agents, were consistently associated with improvements in the skin microbiome and epidermal barrier integrity. Reduced *S. aureus*colonization was consistently observed across all the skin microbiome studies evaluating dupilumab (N=4), suggesting restoration of microbial balance and innate immune defense [21, 55, 63, 69]. A similar result was found for tralokinumab, although current evidence is limited to a single study [77]. Notably, changes in skin microbiome associated to dupilumab were also correlated with improvement in clinical scales (i.e., EASI score) (Table 1) [21]. Kenney et al. analyzed ceramide synthase 1 (*CERS1*) expression as a marker of *S. aureus* colonization and impaired long-chain fatty acid synthesis, finding significant post-dupilumab reductions in *CERS1* expression and *S. aureus*colonization, reflecting improved lipid barrier recovery together with microbial normalization [63].

TEWL and stratum corneum hydration (SCH) were also evaluated as barrier function markers. Montero et al. assessed their predictive value for dupilumab response, showing that SCH changes in both lesional and non-lesional skin after 16 weeks could serve as early predictors of sustained treatment response [50]. Comparable microbiome and barrier function analyses remain limited for JAK inhibitors and emerging therapies, highlighting a current gap in comparative data.

### Risk of Bias

 Methodologically, most studies presented a moderate risk of bias, primarily due to the absence of control groups, potential confounders, or loss to follow-up. A minority were rated as high risk owing to small sample size and/or limited methodological detail. Clinical trials were classified as having low risk of bias, given their more rigorous design and clearer methodology, which minimized these limitations.

## Discussion

**Fig. 3:**
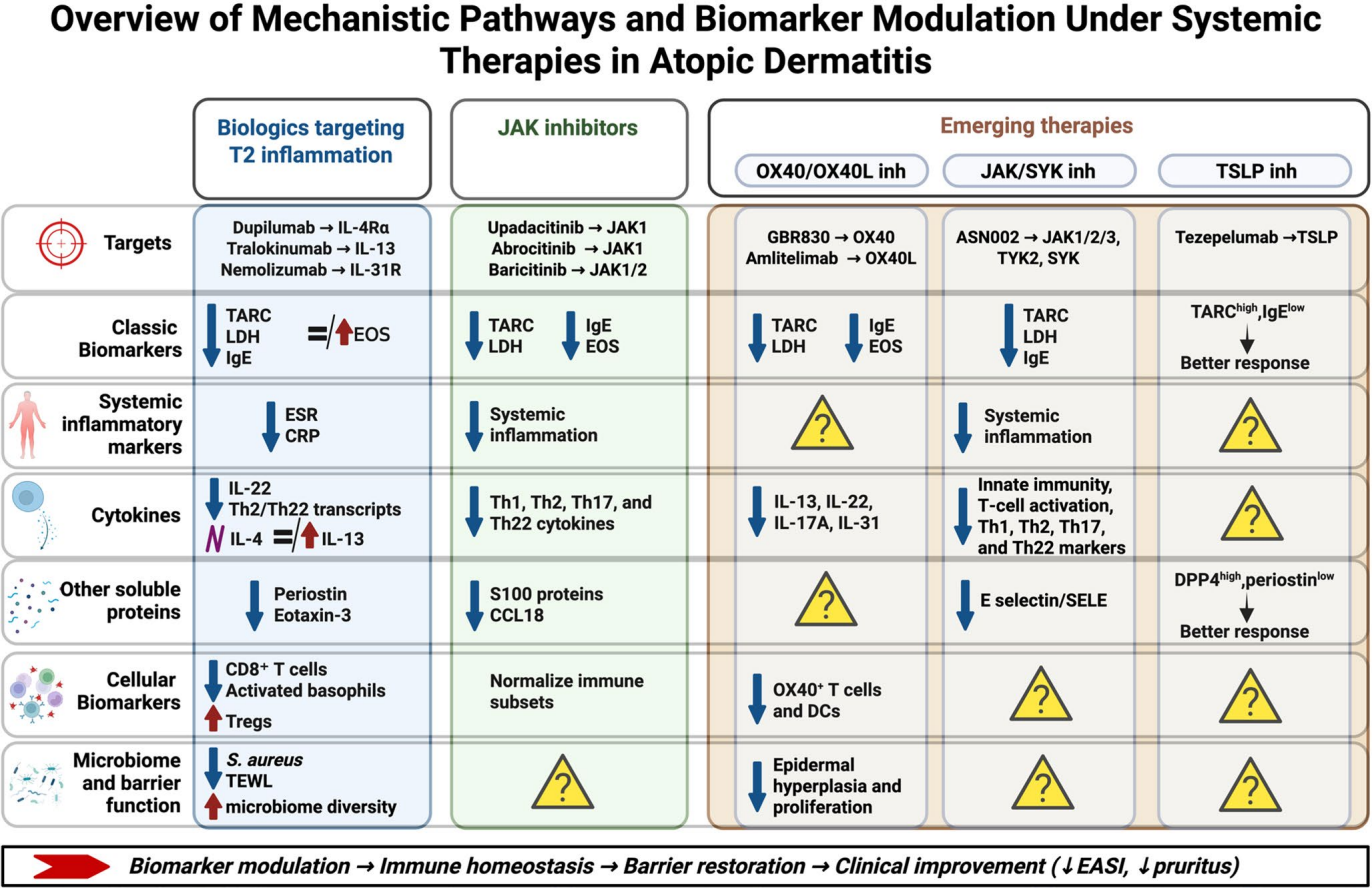
Biomarker changes across therapeutic classes included in the review**.** The direction and consistency of biomarker changes across therapeutic classes is shown, illustrating the pathway from biomarker modulation to immune homeostasis, barrier recovery, and clinical remission

 Systemic therapies for AD act through distinct immunologic mechanisms, ranging from extracellular cytokine blockade with biologics to intracellular JAK–STAT inhibition and emerging upstream modulators (OX40/OX40L, TSLP) (Figure 2). Collectively, these interventions converge toward a common endpoint: restoration of immune balance, epidermal barrier repair, and clinical remission. Our systematic review suggests that biologic therapies and JAK inhibitors consistently modulate key biomarkers such as CCL17, LDH, and IgE, generally correlating with clinical improvement. Transcriptomic and proteomic analyses indicate normalization of inflammatory profiles and restoration of skin barrier function following these treatments [15–94], although current evidence is scarce. Changes in skin microbiome, with specific reductions in*S. aureus* colonization were also associated to the different therapies. Figure 3 summarizes the direction and consistency of biomarker changes across therapeutic classes, illustrating the pathway from biomarker modulation to immune homeostasis, barrier recovery, and clinical remission.

In recent years, the search for biomarkers capable of reflecting disease severity and predicting therapeutic response has emerged as a research priority in AD. To date, CCL17 is considered the most reliable serum biomarker associated with AD severity and disease course [7–9]. CCL17 activity plays a central role in AD pathogenesis, as this Th2-associated chemokine recruits T cells to the skin, making it a key pathogenic mediator and therapeutic target [7–9]. In our review, CCL17 was the most frequently studied biomarker (reported in over 40 studies), showing consistent decreases after treatment regardless of the drug class (60-85% reduction), and strong correlations with improvement in clinical scores (i.e., EASI and SCORAD). However, its applicability is limited by interindividual variability (influenced by age) and non-specific elevations in other inflammatory conditions [7–9]. To our knowledge, routine measurement of serum CCL17 is only implemented in Japan, where is used as clinical marker to monitor disease activity and even treatment response [7–9]. Alongside CCL17, LDH, is among the most frequently assessed biomarkers, and its levels decrease independently of the treatment used. Interestingly, in the case of dupilumab studies, LDH reduction correlated with improved EASI scores, whereas elevated baseline levels may predict suboptimal treatment response [18, 25, 27, 29, 31, 39, 53, 59, 66]. However, the main limitation of this marker is the lack of disease specificity, limiting its standalone predictive value [7, 9]. Total serum IgE, for its part, has been evaluated as a potential marker of disease severity, although elevations are not uniform across all patients and are mainly observed in those with an allergic comorbidity. Most studies included in our review reported a reduction in IgE following dupilumab treatment. Tralokinumab, upadacitinib, and several investigational agents (AKN120, amlitelimab, and stapokibart) also showed similar evidence [74–76, 82, 85, 86, 92–94]. Reduction of specific IgE against certain aeroallergens in patients receiving dupilumab, either as monotherapy or combined with immunotherapy, suggest immune tolerance induction and supports the drug’s utility in the comprehensive management of AD and associated comorbidities [65].

Cytokine profiling highlighted mechanistic distinctions between drug classes. Dupilumab primarily targets Th2-mediated signaling via IL-4Rα blockade, but its immunologic effects extend beyond the Th2 axis. While some Th2-associated cytokines (IL-4, IL-13; probably reflecting receptor blocking) and also eosinophils may paradoxically increase in circulation [32, 39, 41, 47, 58], reductions in downstream mediators such as IL-12, IL-22, TNF𝛼, IL-1b, IL-6, IL-17A and other inflammatory molecules indicate broader network modulation [24, 47, 57, 58]. JAK inhibitors exert broader immunomodulatory effects across Th1, Th2, Th17, and Th22 axes, reflected by reductions in IL-4, IL-13, IL-9, IL-17A, IL-22, IFN-γ, TNF-α [84]. Emerging therapies (e.g., OX40/OX40L inhibitors, TSLP blockade) target upstream signaling nodes, producing overlapping downstream reductions in Th2-associated transcripts, although clinical data remain limited.

The introduction of “omics” technologies has contributed to refine this picture, identifying key alterations in skin barrier function, immune profiles, and the cutaneous microbiome, and laying the groundwork for novel biomarker discovery and personalized therapies [95, 96]. Transcriptomic analyses of both skin biopsies and less invasive tape-strip samples have revealed consistent gene expression patterns associated with Th2 and Th22 inflammation and barrier dysfunction in AD [95, 96]. Proteomic and lipidomic studies have demonstrated changes in barrier proteins and lipid composition, highlighting their modulatory role in cutaneous inflammatory responses [95, 96]. The impact of innovative therapies on these molecular and cellular levels has been assessed in several studies included in our review. Transcriptomic analyses of dupilumab, tralokinumab, abrocitinib, GBR830, and ASN002 demonstrate that these drugs can modulate the molecular signature of AD [15, 16, 30, 76, 79, 87, 89]. They reduce expression of Th2 inflammation-related genes as early as four weeks of treatment [89]. In the case of tralokinumab, gene expression related to Th cells and systemic inflammation (including atherosclerosis and vascular inflammation) continues to be modulated after two years, achieving a profile similar to non-lesional skin [76]. Simultaneously, epidermal proliferation is reduced, and genes involved in lipid metabolism and epidermal cohesion are upregulated, progressively restoring barrier function [76]. Of particular interest is the progressive normalization of barrier-related transcripts following systemic therapy: for example, dupilumab has been associated with upregulation of barrier and lipid-metabolism genes (periplakin, FA2H, LOR, CLDN8, FLG, KRT1, KRT10, and ELOVL3) [30, 32], tralokinumab with restoration of CLDN1 and LOR expression [76], and JAK/SYN inhibitor with increased FLG and CLDN genes [89]. Proteomic analyses confirm significant reductions in key inflammatory markers, and metabolomic studies demonstrate notable changes in lipid composition with these new therapies [24, 49, 68]. Miyamato et al. identified ribose as biomarker for predicting efficacy of dupilumab [49].

 The skin microbiome is increasingly recognized as a dynamic biomarker of treatment response. Epidermal barrier dysfunction promotes *S. aureus*proliferation, which in turn helps maintain a proinflammatory Th2 microenvironment [1, 2, 4, 7]. Dupilumab and tralokinumab reduce*S. aureus *colonization and increase microbial diversity from the first weeks of treatment [55, 69, 77]. These effects correlate with decreases in CCL17/CCL18 and improvements in EASI and SCORAD [55]. Mechanistically, this outcome may result from stimulation of the innate and IL-17-mediated immune response, which activates neutrophils and facilitates*S. aureus*clearance via lysozyme and complement pathways [55].

While these molecular, immunologic, and microbial findings illuminate common therapeutic pathways, it is increasingly clear that biomarker behavior is shaped by underlying disease endotypes [97, 98]. Distinct biomarker profiles have been characterized across clinically relevant endotypes of AD, underscoring the marked immunological and molecular heterogeneity of the disease. The extrinsic endotype (the most prevalent form), is defined by elevated total and specific IgE levels, eosinophilia, and robust Th2-driven inflammation, with increased expression of IL-4, IL-5, IL-13, and chemokines such as CCL17 [97, 98]. In contrast, intrinsic AD typically exhibits normal IgE levels, absence of sensitization, and a more prominent Th1/Th17/Th22 activation pattern, including higher levels of IFN-γ, IL-17, and IL-22, suggesting distinct molecular drivers despite comparable clinical severity [97, 98]. Ethnic background also contributes to biomarker variability. Asian AD is associated with augmented Th17 and Th22 signatures and marked epidermal hyperplasia, whereas AD in individuals of African ancestry demonstrates enhanced Th2/Th22 polarization and reduced FLG expression, often independent of FLG mutations. The Caucasian AD endotype typically exhibits a predominantly Th2-driven inflammatory profile, with variable but generally moderate involvement of Th22 and Th1 pathways. This immunologic pattern occurs in parallel with a higher prevalence of FLG loss-of-function mutations in this population [97, 98]. Age of onset further shapes the immunological landscape. Adult-onset AD shows broad activation across Th2 (IL-13, IL-31, CCL17), Th22 (IL-22, S100 proteins), Th17 (IL-17A, IL-19, CCL20, LL-37, PI3/elafin), and Th1 (IFN-γ, CXCL9-11) pathways. Conversely, pediatric AD exhibits attenuated Th1 activity but increased Th9 (IL-9) responses, heightened innate immunity (IL-1β, IL-8, IFN-α1), and pronounced lipid metabolic dysfunction contributing to early epidermal barrier impairment [97, 98]. Collectively, these endotype variations not only reflect the pathophysiological diversity of AD but may also modulate biomarker trajectories and influence differential responses to targeted therapies. Indeed, phenotype-specific variations in treatment outcomes have been reported; a multicenter study by Nettis et al. including 543 adults found that patients with non-classic adult-type AD phenotypes responded slightly better to dupilumab than those with classic flexural disease [38].

Despite compelling evidence, the main limitation of this systematic review is the heterogeneity of included studies in terms of design, sample size, biomarkers evaluated, and analytical techniques. Most publications presented a moderate risk of bias. Dupilumab is the most extensively studied drug, as it was the first approved, while evidence for other biologics and JAK inhibitors remains limited, restricting the generalizability of findings. Variability in the timing of measurements and differences in analytical methods further complicate direct comparisons across studies. Additionally, the correlation between biomarker changes and clinical outcomes, a key aspect for identifying predictors of therapeutic response, was insufficiently explored in most studies. Although we observed associations with several cytokines and microbiome features, it should be noted that most of these markers require research-level platforms and are not yet validated for clinical decision-making. Furthermore, the vast majority of data is derived from adult cohorts, and the evidence for pediatric, adolescent or elderly patients is scarce. Moreover, across the 80 included studies, many enrolled mixed AD endotypes without clearly specifying intrinsic versus extrinsic forms, while others did not identify the phenotype at all. Ethnic representation was uneven, with most participants being Caucasian, limiting applicability to other populations. Several studies combined adult-onset and pediatric-onset AD, and although most focused on adults, some included children and adolescents. These variations in endotype, ethnicity, and age likely influence baseline biomarker profiles, affecting IgE, eosinophils, CCL17, IL-22, Th17/Th22 cytokines, lipid metabolism, and microbiome composition, and may contribute to the variability in treatment-associated biomarker changes observed across studies. The 10-year temporal window, potential publication bias, and exclusion of non-indexed studies are also limitations. Given the rapid evolution of this field, recent publications may not be included in this review. Finally, clinicians should also be aware of emerging reports of phenotypic switching from AD to psoriasis in some patients receiving biologics or JAK inhibitors [99–101], underscoring the need for careful monitoring during therapy.

 The integration of omics technologies into AD research has marked a turning point, enabling deeper understanding of pathophysiology and the development of therapies that have transformed disease management and patient QoL. Despite the proven efficacy of current treatments, validated biomarkers for objectively assessing disease severity and predicting therapeutic response are lacking. Although CCL17 and LDH remain the most consistent candidates, it is unlikely that any single biomarker will fully meet these needs. Future strategies are expected to rely on combined panels integrating immunologic, transcriptomic, and microbiome data, facilitating more precise patient stratification and advancing personalized medicine. Multicenter studies with large cohorts and standardized techniques will be essential to confirm the clinical and predictive value of these biomarker panels.

## Supplementary Information

Below is the link to the electronic supplementary material.

Supplementary Material 1 (DOCX 17.2 KB)

## Data Availability

All data supporting the findings of this study are available within the paper and its Supplementary Information.
