# Supplementary material for: Novel Therapeutic Strategies for Atopic Dermatitis: Biomarker Modulation and Clinical Implications. A Systematic Review
Source: Clin Rev Allergy Immunol. 2026 Jan 29;69(1):4. doi: 10.1007/s12016-025-09129-z (PMC12855363; doi:10.1007/s12016-025-09129-z)
Supplement: Supplementary file 1 — Supplementary Material 1 (DOCX 17.2 KB) [file 12016_2025_9129_MOESM1_ESM.docx]

Database: PubMed

Search date: January 17, 2025

Results: 291

Search Strategy

#1 ((((((((((((((((((((((((((((((((((((((((((((((("Biological Products"[Mesh]) OR (Biologic Product[Text Word])) OR (Product, Biologic[Text Word])) OR (Products, Biological[Text Word])) OR (Biologic Products[Text Word])) OR (Biological Product[Text Word])) OR (Product, Biological[Text Word])) OR (Biopharmaceuticals[Text Word])) OR (Biopharmaceutical[Text Word])) OR (Biological Drug[Text Word])) OR (Drug, Biological[Text Word])) OR (Biologic Drugs[Text Word])) OR (Drugs, Biologic[Text Word])) OR (Biological Drugs[Text Word])) OR (Drugs, Biological[Text Word])) OR (Biological Medicines[Text Word])) OR (Medicines, Biological[Text Word])) OR (Biologicals[Text Word])) OR (Biologic Medicines[Text Word])) OR (Medicines, Biologic[Text Word])) OR (Biologic Pharmaceuticals[Text Word])) OR (Pharmaceuticals, Biologic[Text Word])) OR (Biologics[Text Word])) OR (Biologic Drug[Text Word])) OR (Drug, Biologic[Text Word])) OR (Biological Medicine[Text Word])) OR (Medicine, Biological[Text Word])) OR (dupilumab[Text Word])) OR (Tralokinumab[Text Word])) OR (lebrikizumab[Text Word])) OR ("Janus Kinases"[Mesh])) OR (Kinases, Janus[Text Word])) OR (Janus Kinase[Text Word])) OR (Kinase, Janus[Text Word])) OR (JAK Kinases[Text Word])) OR (JAK Kinase[Text Word])) OR (Kinases JAK[Text Word])) OR (upadacitinib[Text Word])) OR (abrocitinib[Text Word])) OR (baricitinib[Text Word])) OR (OX40)) OR (rocatinlimab)) OR (telazorlimab)) OR (stapokibart)) OR (amlitelimab)) OR (nemolizumab)) OR (rezpegaldesleukin))

#2 (((((((((((((((((((((((((((((((((((("Biomarkers"[Mesh]) OR (marker*[Text Word]) OR (Biomarker[Text Word])) OR (Marker, Biological[Text Word])) OR (Biological Marker*[Text Word])) OR (Markers, Biological[Text Word])) OR (Biological Markers[Text Word])) OR (Biologic Marker*[Text Word])) OR (Markers, Biologic[Text Word])) OR (Biologic Marker[Text Word])) OR (Marker, Biologic[Text Word])) OR (Surrogate Markers[Text Word])) OR (Marker, Surrogate[Text Word])) OR (Surrogate Marker[Text Word])) OR (Markers, Surrogate[Text Word])) OR (Markers, Immunologic[Text Word])) OR (Immune Marker[Text Word])) OR (Marker, Immune[Text Word])) OR (Immune Markers[Text Word])) OR (Markers, Immune[Text Word])) OR (Immunologic Marker[Text Word])) OR (Marker, Immunologic[Text Word])) OR (Immunologic Markers[Text Word])) OR (Markers, Laboratory[Text Word])) OR (Laboratory Marker[Text Word])) OR (Marker, Laboratory[Text Word])) OR (Laboratory Markers[Text Word])) OR (Serum Markers[Text Word])) OR (Marker, Serum[Text Word])) OR (Serum Marker[Text Word])) OR (Markers, Serum[Text Word])) OR (Biochemical Marker[Text Word])) OR (Marker, Biochemical[Text Word])) OR (Biochemical Markers[Text Word])) OR (Markers, Biochemical[Text Word])) OR (biomarker*)) OR ("biomarker s"))

#3 ((((("Dermatitis, Atopic"[Mesh]) OR (Atopic Dermatitis[Text Word])) OR (Atopic Eczema[Text Word])) OR (atopic neurodermatitis[Text Word])) OR ("eczema atopic"[tiab:~3] OR eczema* dermatitis[Text Word]))

#1 AND #2 AND #3

Filters applied: publication date from 2014 to 2024.

The search was developed using a combination of Medical Subject Headings (MeSH) and free-text terms to capture a comprehensive set of relevant studies. This approach ensures the inclusion of articles that may not be yet indexed with MeSH terms.

Database: Embase (OVID) <1974 to 2025 January 16>

Search date: January 17, 2025

Results: 251

Search Strategy

1 biological product/

2 (biological adj3 product*).mp.

3 (biological adj3 agent*).mp.

4 biopharmaceutical*.mp.

5 (biological adj3 drug*).mp.

6 (biological adj3 medicine*).mp.

7 dupilumab.mp.

8 Tralokinumab.mp.

9 lebrikizumab.mp.

10 Janus kinase/

11 (janus adj3 kinase*).mp.

12 (jak adj3 kinase*).mp.

13 upadacitinib.mp.

14 abrocitinib.mp.

15 baricitinib.mp.

16 OX40.mp.

17 rocatinlimab.mp.

18 telazorlimab.mp.

19 stapokibart.mp.

20 amlitelimab.mp.

21 nemolizumab.mp.

22 rezpegaldesleukin.mp.

23 1 or 2 or 3 or 4 or 5 or 6 or 7 or 8 or 9 or 10 or 11 or 12 or 13 or 14 or 15 or 16 or 17 or 18 or 19 or 20 or 21 or 22

24 biological marker/

25 biomarker*.mp.

26 (biological adj3 marker*).mp.

27 (marker adj3 biologic*).mp.

28 (surrogate adj3 marker*).mp.

29 (immunological adj3 marker*).mp.

30 (laboratory adj3 marker*).mp.

31 (serum adj3 marker*).mp.

32 (biochemical adj3 marker*).mp.

33 biomarker s.mp.

34 atopic dermatitis/

35 atopic dermatitis.mp.

36 atopic eczema.mp.

37 atopic neurodermatitis.mp.

38 (eczema adj3 atopic).mp.

39 (eczema adj3 dermatitis).mp.

40 34 or 35 or 36 or 37 or 38 or 39

41 24 or 25 or 26 or 27 or 28 or 29 or 30 or 31 or 32 or 33

42 23 and 40 and 41

43 limit 42 to embase status

Filters applied: publication date from 2014 to 2024.

The search strategy combined Emtree controlled vocabulary terms with free-text keywords to increase sensitivity and retrieve all relevant literature on the topic.

Database: Scopus

Search date: January 17, 2025

Results: 203

Search Strategy

( ( TITLE-ABS-KEY ( biological W/3 product* ) ) OR ( TITLE-ABS-KEY ( biological W/3 agent* ) ) OR ( TITLE-ABS-KEY ( biopharmaceutical* ) ) OR ( TITLE-ABS-KEY ( biological W/3 agent* ) ) OR ( TITLE-ABS-KEY ( biological W/3 medicine* ) ) OR ( TITLE-ABS-KEY ( dupilumab ) ) OR ( TITLE-ABS-KEY ( tralokinumab ) ) OR ( TITLE-ABS-KEY ( lebrikizumab ) ) OR ( TITLE-ABS-KEY ( janus W/3 kinase* ) ) OR ( TITLE-ABS-KEY ( jak W/3 kinase* ) ) OR ( TITLE-ABS-KEY ( upadacitinib ) ) OR ( TITLE-ABS-KEY ( abrocitinib ) ) OR ( TITLE-ABS-KEY ( baricitinib ) ) OR ( TITLE-ABS-KEY ( ox40 ) ) OR ( TITLE-ABS-KEY ( rocatinlimab ) ) OR ( TITLE-ABS-KEY ( telazorlimab ) ) OR ( TITLE-ABS-KEY ( stapokibart ) ) OR ( TITLE-ABS-KEY ( amlitelimab ) ) OR ( TITLE-ABS-KEY ( nemolizumab ) ) OR ( TITLE-ABS-KEY ( rezpegaldesleukin ) ) ) AND ( ( TITLE-ABS-KEY ( ( biomarker AND s ) ) ) OR ( TITLE-ABS-KEY ( biochemical W/3 marker* ) ) OR ( TITLE-ABS-KEY ( serum W/3 marker* ) ) OR ( TITLE-ABS-KEY ( laboratory W/3 marker* ) ) OR ( TITLE-ABS-KEY ( immunological W/3 marker* ) ) OR ( TITLE-ABS-KEY ( surrogate W/3 marker* ) ) OR ( TITLE-ABS-KEY ( marker W/3 biologic* ) ) OR ( TITLE-ABS-KEY ( biological W/3 marker* ) ) ) AND ( ( TITLE-ABS-KEY ( atopic W/3 eczema ) ) OR ( TITLE-ABS-KEY ( atopic W/3 neurodermatitis ) ) OR ( TITLE-ABS-KEY ( ( atopic W/3 dermatitis ) ) ) )

Filters applied: publication date from 2014 to 2024.

Database: Web of Science Core Collection

Search date: January 17, 2025

Results: 209

Search Strategy

#1 TS=((biological NEAR/3 product*))

#2 TS=((biological NEAR/3 agent*))

#3 TS=((biopharmaceutical))

#4 TS=((biological NEAR/3 medicine*))

#5 TS=((dupilumab))

#6 TS=((Tralokinumab))

#7 TS=((lebrikizumab))

#8 TS=((janus NEAR/3 kinase*))

#9 TS=((jak NEAR/3 kinase*))

#10 TS=((upadacitinib))

#11 TS=((abrocitinib))

#12 TS=((baricitinib))

#13 TS=((OX40))

#14 TS=((rocatinlimab))

#15 TS=((telazorlimab))

#16 TS=((stapokibart))

#17 TS=((amlitelimab))

#18 TS=((nemolizumab))

#19 TS=((rezpegaldesleukin))

#20 #19 OR #18 OR #17 OR #16 OR #15 OR #14 OR #13 OR #12 OR #11 OR #10 OR #9 OR #8 OR #7 OR #6 OR #5 OR #4 OR #3 OR #2 OR #1

#21 TS=(biological NEAR/3 marker*)

#22 TS=(marker NEAR/3 biologic*)

#23 TS=(surrogate NEAR/3 marker*)

#24 TS=(immunological NEAR/3 marker*)

#25 TS=(laboratory NEAR/3 marker*)

#26 TS=(serum NEAR/3 marker*)

#27 TS=(biochemical NEAR/3 marker*)

#28 TS=(biomarker s)

#29 TS=(biomarker*)

#30 #29 OR #28 OR #27 OR #26 OR #25 OR #24 OR #23 OR #22 OR #21

#31 TS=(atopic NEAR/3 dermatitis)

#32 TS=(atopic NEAR/3 eczema)

#33 TS=(atopic NEAR/3 neurodermatitis)

#34 #33 OR #32 OR #31

#35 #34 AND #30 AND #20

Filters applied: publication date from 2014 to 2024.
